# Preventive effect of sodium bicarbonate Ringer’s solution on post-contrast acute kidney injury in patients undergoing percutaneous coronary intervention

**DOI:** 10.3389/fphar.2025.1542776

**Published:** 2025-09-05

**Authors:** Jialin Liu, Wen Zhao, Baobao Feng, Chong Meng, Deya Shang

**Affiliations:** ^1^ Department of Emergency, Shandong Provincial Hospital Affiliated to Shandong First Medical University, Jinan, China; ^2^ Department of Poisoning and Occupational Diseases, Shandong Provincial Hospital Affiliated to Shandong First Medical University, Jinan, China

**Keywords:** sodium bicarbonate Ringer’s solution, contrast, post-contrast acute kidney injury, hydration, coronary angiography, percutaneous coronary interventions

## Abstract

**Background:**

Post-contrast acute kidney injury (PC-AKI) is a common complication of coronary angiography (CAG) and percutaneous coronary interventions (PCI). Sodium bicarbonate Ringer’s solution (BRS) has been shown to reduce the incidence of postoperative AKI and the risk of acid-base disorder in surgical and critically ill patients. There is no research data of BRS in the field of PC-AKI. Therefore, this study aimed to evaluate the efficacy and safety of BRS in the prevention of PC-AKI.

**Methods:**

Patients with coronary atherosclerotic heart disease and stage 2–3b chronic kidney disease (CKD) who underwent CAG or PCI were prospectively enrolled and randomly assigned to BRS group or normal saline (NS) group from February 2024 to October 2024. The patients were given BRS or NS for hydration at 1mL/kg/h from 3 h before to 4 h after CAG/PCI. Serum creatinine (Scr), cystatin C (Cys-C) and neutrophil gelatinase-associated apolipoprotein (NGAL) were measured within 3 days before and 48 h after iodinated contrast agent exposure. The primary endpoint was PC-AKI, and the secondary endpoints were the need of renal replacement therapy (RRT) and major adverse cardiovascular events (MACEs) within 30 days after CAG/PCI. The correlation between BRS and PC-AKI was analyzed by multivariate logistic regression.

**Results:**

The baseline characteristics of patients in the two groups were similar, and the changes of Cys-C, NGAL, and electrolytes before and after CAG/PCI were not statistically significant. PC-AKI occurred in 1 patient (1.3%) in BRS group and 7 patients (9.3%) in NS group, and the difference was statistically significant (*P* = 0.029). Multivariate logistic regression showed that there was an approaching statistical trend of BRS to reduce the incidence of PC-AKI (*P* = 0.054).

**Conclusion:**

BRS might be more suitable than NS in reducing the incidence of PC-AKI for patients with mild-to-moderate renal dysfunction who underwent CAG/PCI.

## 1 Introduction

There are more than 11 million patients with coronary atherosclerotic heart disease (CAD) in China. Post-contrast acute kidney injury (PC-AKI) has become a common postoperative complication with the extensive application of coronary angiography (CAG) and percutaneous coronary intervention (PCI). The incidences of PC-AKI after PCI were reported to range from 0% to 24% in previous researches (Chen et al., 2008; Berwanger et al., 2011; Bolognese et al., 2012). Renal insufficiency is the most important risk factors associated with PC-AKI (Tepel et al., 2000). Contrast exposure has been avoided in patients with severe chronic kidney disease (CKD) (glomerular filtration rate (GFR) < 45 mL/min). Patients with CKD undergoing CAG or PCI are at high risk of PC-AKI and have a 3-fold increased risk of mortality, thus they need active prevention of PC-AKI (Silvain et al., 2018; van der Molen et al., 2018a).

Hydration has been the main preventive measure for PC-AKI in patients exposed to contrast. However, there were no high-evidenced recommendations on hydration scheme and hydration fluids. Normal saline (NS) is widely used to prevent PC-AKI, but it has been observed to result in hyperchloric acidosis (Yunos et al., 2012; Semler and Rice, 2016). Sodium bicarbonate solution drew researchers’ attention due to its effect on acidosis. Some clinical trials demonstrated that sodium bicarbonate solution was more effective than NS in prevention of contrast-induced nephropathy (Merten et al., 2004; Masuda et al., 2007; Ozcan et al., 2007), which was not consistent with following studies (Adolph et al., 2008; Brar et al., 2008; Boucek et al., 2013). The inconsistence might be partially explained by the heterogeneity between study design and cohorts. There was risk of acid-base disturbance when sodium bicarbonate solution with higher concentration than physiological concentration was used, while a lower concentration was difficult to prepare and store. Based on the above, neither NS nor sodium bicarbonate solution was ideal hydration solution for preventing PC-AKI. Sodium bicarbonate Ringer’s solution (BRS) is a new type of crystalloid solution containing an appropriate amount of electrolytes and bicarbonate (Na^+^ 130 mmol/L, K^+^ 4.0 mmol/L, Ca^2+^ 1.5 mmol/L, Mg^2+^ 1.0 mmol/L, Cl^−^ 109 mmol/L, HCO_3_^−^ 28 mmol/L, and Citrate^3−^ 1.3 mmol/L) with an osmotic pressure of 240–275 mOsm/(kg▪H_2_0), which is more closer to the normal physiological environment. Animal experiments and phase I/II clinical studies have explained the pharmacological mechanism and pathophysiological effects of BRS to some extent (Satoh et al., 2005; Pakfetrat et al., 2019). BRS has been confirmed to reduce the incidence of postoperative AKI in surgical patients and the risk of acid-base imbalance (Wang et al., 2021; Wu et al., 2022). And it also showed a potential renal function protective effect in critically ill patients, and a preventive effect on cisplatin-induced AKI in patients with esophageal cancer, compared with NS (Bian et al., 2022; Takemura et al., 2025). Theoretically, BRS could avoid the shortages of NS and sodium bicarbonate solution, and may be a better choice for the prevention of PC-AKI. So far, there are few studies focusing on the effect of hydration with BRS to prevent PC-AKI. Based on a similar pathophysiology, we hypothesized that BRS may have a preventive effect of PC-AKI in patients undergoing CAG or PCI. Therefore, we conducted this study to compare the effects of hydration with BRS or NS on the incidence of PC-AKI after CAG or PCI in patients with mild to moderate CKD, aiming to explore the feasibility of using BRS for hydration therapy in clinical practice.

## 2 Methods

### 2.1 Design, setting, and participants

This was a single-center prospective randomized controlled study. Patients with CAD and CKD stage 2–3b who were hospitalized and underwent CAG or PCI in the department of emergency, Shandong Provincial Hospital were enrolled from February 2024 to October 2024. The study was approved by the biomedical research ethic committee of Shandong Provincial Hospital Affiliated to Shandong First Medical University (SWYX:NO. 2021-085) and registered in Chinese Clinical Trial Registry (https://www.chictr.org.cn/) with the registration number of ChiCTR2400079751.

Patients were included if they fulfilled all the following criteria: (1) ≥ 18 years; (2) diagnosis with CAD and scheduled for CAG or PCI; (3) mild-to-moderate renal dysfunction in CKD stage 2–3b; (4) cardiac function in grade I-II (Killip or New York heart association (NYHA) classification]; (5) informed consents were obtained from patients or their guardians.

Patients were excluded if they met one of the following criteria: (1) usage of intravascular contrast within 72 h; (2) estimated glomerular filtration rate (eGFR) < 15 mL/min/1.73 m^2^ or requiring dialysis; (3) hypotension (systolic blood pressure <90 mmHg) or hemodynamic instability; (4) severe valvular heart disease; (5) history of organ transplantation or malignant tumor; (6) contrast allergy; (7) liver dysfunction and coagulation disorders; (8) infection; (9) acid-base imbalance and electrolyte disorder; (10) use of metformin within 48 h; (11) nephrotoxic drugs, such as chemotherapy drugs, non-steroidal anti-inflammatory drugs (except aspirin), amphotericin, aminoglycosides, *etc.*, Within 1 month; (12) pregnant or lactating women; (13) abnormal thyroid function. These criteria were made based on previous research.

### 2.2 Randomization and hydration protocol

Patients were randomized using a computer-generated randomization code and the sealed envelope method. One hundred and sixty numbers were randomized into the BRS group or NS group in a 1:1 ratio by computer-generated randomization code, then they were sealed in opaque envelopes with disrupted order. Consecutively participants were enrolled in BRS or NS group according to the hydration regimen corresponding to the number insides the envelopes. Physician A was responsible for the recruitment and randomization of patients. The hydration programme was carried out by an expertized nurse. Physician B who were blinded for the hydration groups collected participants’ demographic and treatment data.

All patients underwent CAG or PCI via radial artery, and isotonic contrast (iodixanol) was used during the operation. All patients were treated with dual antiplatelets (aspirin + clopidogrel/ticagrelor) and statins before and after PCI according to the guidelines [20]. Patients from the two groups received continuous intravenous infusion of BRS (Jiangsu Hengrui Pharmaceuticals Co., Ltd., China) or 0.9% NS (Chenxin Pharmaceuticals Co., Ltd., China) for hydration at a rate of 1.0 mL/kg/h (0.5 mL/kg/h if left ventricular ejection fraction (LVEF) ≤35%) from 3 h before to 4 h after PCI, based on recommendations of the 2018 European Society of Urogenital Radiology (ESUR) guidelines [21].

### 2.3 Data collection

The baseline characteristics of all enrolled patients were recorded, including gender, age, body mass index (BMI), tobacco and alcohol consumption, previous diseases (hypertension, diabetes, etc.). Diabetes can be diagnosed based on typical symptoms (as excessive thirst, frequent urination, excessive hunger, and unexplained weight loss) and one of the following: (1) fasting venous glucose ≥7.0 mmol/L, (2) the 2-h venous glucose in the oral glucose tolerance test (OGTT) ≥ 11.1 mmol/L, (3) HbA1c ≥ 6.5%, (4) the random venous glucose ≥11.1 mmol/L. If typical symptoms are lacking, two indicators at the same time-point or the same indicator at two different time-points reach or exceed the cut-off point (excluding random blood glucose), then the diagnosis of diabetes can be made. Hypertension is defined as the blood pressure ≥140/90 mmHg measured at three time-points of different days, without the use of antihypertensive drugs. Serological indicators after admission were collected, such as high-density lipoprotein (HDL), low-density lipoprotein (LDL), total cholesterol (TC), hemoglobin (Hb), electrolytes (potassium, sodium, chloride), carbon dioxide combining power, etc. The dosages of contrast used during CAG or PCI were also recorded.

The levels of serum creatinine (Scr), cystatin C (Cys-C) and neutrophil gelatinase-associated apolipoprotein (NGAL) were collected within 3 days before and 48 h after exposure to contrast in both groups. The eGFR was calculated according to the following formula:

Female Scr ≤62 μmol/L: $\text{eGFR}=144\times {\left(\frac{\text{sCr}}{62}\right)}^{-0.329}\times 0.993^{\text{Ag}\mathrm{e}}$

Female Scr >62 μmol/L: $\text{eGFR}=144\times {\left(\frac{\text{sCr}}{62}\right)}^{-1.209}\times 0.993^{\text{Ag}\mathrm{e}}$

Male Scr ≤80 μmol/L: $\text{eGFR}=141\times {\left(\frac{\text{sCr}}{80}\right)}^{-0.411}\times 0.993^{\text{Ag}\mathrm{e}}$

Male Scr >80 μmol/L: $\text{eGFR}=141\times {\left(\frac{\text{sCr}}{80}\right)}^{-1.209}\times 0.993^{\text{Ag}\mathrm{e}}$

### 2.4 Endpoints

The primary endpoint was PC-AKI, defined as an increase in Scr of ≥0.3 mg/dL (26.52 μmol/L) or more than 50% from the baseline within 48 h after exposure to contrast according to the 2018 ESUR guidelines [6]. The secondary end points were persistent renal dysfunction requiring continuous renal replacement therapy (CRRT) and major adverse cardiovascular events (MACEs) within 30 days after CAG/PCI. MACE were defined as death related to CAD, non-fatal myocardial infarction, unstable angina, heart failure, fatal or non-fatal ischemic stroke, and re-admissions due to the above conditions.

### 2.5 Statistical analysis

Shapiro-Wilk test was used to test the normality of continuous variables. Normal distributed variables were described by mean ± standard deviation, while non-normal distributed variables were described by median and inter-quartile range (IQR). Student’s t-test or Mann-Whitney U test was used to compare the differences in demographic characteristics between the two groups. Categorical variables were expressed as frequencies and percentages, and differences between groups were compared using the chi-square test or Fisher’s exact test. Univariate logistic regression analysis was performed to evaluate the relationships between variables and primary endpoints. Variables associated with PC-AKI in univariate analysis (P < 0.10) were included in the multivariate model. All statistical analyses were performed using IBM SPSS 25.0 (IBM Corp, Armonk, NY) software. Two-tailed *P* < 0.05 was considered statistically significant.

## 3 Results

One hundred and sixty patients were enrolled, among whom 5 were excluded due to the lack of Scr data of post-operation and 5 were lost to follow-up. Finally, 75 patients were included in the analysis in the BRS and NS group respectively.

### 3.1 Participants’ characteristics

Participants’ characteristics are shown in Table 1. The median age was 69 (61–71) years and 95 (63.3%) were males. Ninety-seven patients had concurrent hypertension and 55 had diabetes. There were no significant differences in demographic characteristics such as age (*P* = 0.831), gender (*P* = 0.611), BMI (*P* = 0.0.737), smoker (*P* = 0.188), and alcoholism (*P* = 0.260). Similarly, no significant differences were found in concurrent medication use and laboratory factors (TC, LDL, HDL, and hemoglobin) between the groups (*P* > 0.05). There were also no differences in the proportion of patients underwent PCI and the doses of contrast between the two groups (*P* > 0.05).

**TABLE 1:** Baseline characteristics of the patients.

Variables	All patients (n = 150)	BRS group (n = 75)	NS group (n = 75)	*P v*alue
Age (years)	69 (61, 71)	69 (60,71)	68 (61,71)	0.831
Male	95 (63.3)	49 (65.3)	46 (61.3)	0.611
BMI (kg/m^2^)	25.41 (23.3, 27.7)	25.15 (23.3, 28.1)	26.12 (23.1, 27.6)	0.737
Smoker	66 (44.0)	37 (49.3)	29 (38.7)	0.188
Alcoholism	38 (25.3)	22 (29.3)	16 (21.3)	0.260
Hypertension	97 (64.7)	50 (66.7)	47 (62.7)	0.608
Diabetes	55 (36.7)	25 (33.3)	30 (40.0)	0.397
Medication				
Metformin	21 (14.0)	9 (12.0)	12 (16.0)	0.480
ACEI/ARB	47 (31.3)	27 (36.0)	20 (26.7)	0.218
Laboratory results				
HDL (mmol/L)	1.1 (1.0, 1.3)	1.1 (0.9, 1.4)	1.1 (0.9, 1.3)	0.755
LDL (mmol/L)	2.5 (2.0, 3.3)	2.5 (1.8, 3.2)	2.5 (2.1, 3.4)	0.183
TC (mmol/L)	4.0 (3.3, 5.0)	3.88 (3.2, 4.9)	4.1 (3.5, 5.3)	0.374
Hemoglobin (g/L)	135 (122, 144)	134 (122, 144)	136 (123, 144)	0.771
PCI	103 (68.7)	54 (72.0)	49 (65.3)	0.379
Contrast dose (mL)	144 (90, 182)	161 (96, 183)	124 (88, 176)	0.104

Data are presented as median (inter-quartile range) or numbers (percentages).

Abbreviations: ACEI/ARB, Angiotensin Converting Enzyme Inhibitors/Angiotensin Receptor Blockers; BMI, body mass index; BRS, Bicarbonate Ringer’s Solution; HDL, high density lipoprotein; LDL, low density lipoprotein; NS, normal saline; PCI, percutaneous coronary intervention; TC, total cholesterol.

### 3.2 Effects of hydration fluids on renal function

The renal function parameters (Scr, Cys-C, NGAL, eGFR) were not significantly different before CAG/PCI between BRS and NS group. At the time-point of 48 h after CAG/PCI, the Scr (82.4 (67.3, 87.2) vs 85.8 (72.5, 99.1), *P* = 0.035) and eGFR (84.0 (76.0, 91.0) vs 76.0 (65.0, 85.0), *P* = 0.001) of BRS group were better that of NS group, while Cys-C and NGAL showed no significant difference (Table 2).

**TABLE 2:** Effects of hydration fluids on renal function parameters.

Variables	All patients (n = 150)	BRS group (n = 75)	NS group (n = 75)	*P* Value
Before CAG/PCI				
Scr (μmol/L)	84.6 (71.9, 91.1)	84.6 (74.3, 90.0)	83.7 (68.7, 95.1)	0.939
Cys-C (mg/L)	1.2 (1.0, 1.3)	1.2 (1.0, 1.3)	1.1 (1.0, 1.3)	0.574
NGAL (ng/mL)	50.9 (40.8, 60.0)	51.9 (40.9, 61.7)	50.4 (40.8, 59.8)	0.772
eGFR (mL/min/1.73m^2^)	80.5 (73.0, 87.0)	82.0 (74.0, 87.0)	80.0 (69.0, 86.0)	0.317
After CAG/PCI				
Scr (μmol/L)	83.1 (70.3, 94.5)	82.4 (67.3, 87.2)	85.8 (72.5, 99.1)	0.035
Cys-C (mg/L)	1.10 (1.00, 1.30)	1.09 (1.00, 1.30)	1.12 (1.01, 1.31)	0.910
NGAL (ng/mL)	53.2 (42.2, 69.8)	53.6 (41.0, 70.2)	52.6 (42.2, 69.5)	0.827
eGFR (mL/min/1.73m^2^)	81.0 (67.8, 88.0)	84.0 (76.0, 91.0)	76.0 (65.0, 85.0)	0.001

Data are presented as median (inter-quartile range).

Abbreviations: BRS, Bicarbonate Ringer’s Solution; CAG, Coronary AngioGraphy; Cys-C, Cystatin C; eGFR, estimated Glomerular Filtration Rate; NGAL, Neutrophil Gelatinase-associated ApoLipoprotein (NGAL); NS, normal saline; PCI, percutaneous coronary intervention; Scr, Serum Creatinine.

### 3.3 Effects of hydration fluids on electrolytes

BRS and NS have a potential effect on potassium, sodium, chloride and carbon dioxide binding power. As shown in Table 3, carbon dioxide binding power before CAG/PCI (25.6 (23.4, 27.2) vs 24.3 (23.2, 26.1), *P* = 0.015) and potassium after CAG/PCI (4.2 (3.9, 4.5) vs 4.1 (3.8, 4.3), *P* = 0.038) were higher in the BRS group, while other indicators showed no significant difference.

**TABLE 3:** Effects of hydration fluids on electrolytes.

Variables	All patients (n = 150)	BRS group (n = 75)	NS group (n = 75)	*P* Value
Before CAG/PCI				
Potassium (mmol/L)	4.1 (3.8,4.3)	4.1 (3.8, 4.3)	4.0 (3.8, 4.2)	0.329
Sodium (mmol/L)	139.5 (138.0, 140.7)	139.5 (138.1, 140.8)	139.4 (138.0.140.6)	0.587
Chloride (mmol/L)	104.9 (102.9, 107.0)	104.8 (103.3, 106.6)	105.0 (102.8, 107.0)	0.991
CO_2_CP (mmol/L)	24.8 (23.4, 26.7)	25.6 (23.4, 27.2)	24.3 (23.2, 26.1)	0.015
After CAG/PCI				
Potassium (mmol/L)	4.1 (3.8, 4.4)	4.2 (3.9, 4.5)	4.1 (3.8, 4.3)	0.038
Sodium (mmol/L)	138.3 (136.6, 139.7)	137.8 (136.0, 139.4)	138.6 (137.0, 139.9)	0.092
Chloride (mmol/L)	104.6 (102.3, 106.8)	105.0 (102.7,106.5)	104.5 (101.6, 107.0)	0.512
CO_2_CP (mmol/L)	24.2 (22.6, 25.8)	24.3 (22.4,85.0)	24.0 (22.6, 25.5)	0.418

Data are presented as median (inter-quartile range).

Abbreviations: BRS, Sodium Bicarbonate Ringer’s Solution; CAG, Coronary AngioGraphy; CO_2_CP, carbon dioxide combining power; NS, normal saline; PCI, percutaneous coronary intervention.

### 3.4 Endpoints

In the study, the primary endpoint (PC-AKI) occurred in 1/75 (1.3%) patient in the BRS group and 7/75 (9.3%) patients in the NS group (*P* = 0.029). No MACEs and severe kidney injury needing CRRT were observed during the follow-up.

### 3.5 Variables associated with PC-AKI

Univariate and multivariate logistic regression analyses were carried out to find factors associated with PC-AKI (Table 4). Only diabetes showed statistical significance (*P* = 0.037) in univariate analysis, and no factors were found to be significantly associated with PC-AKI in multivariate analysis. BRS showed no statistical significance (*P* = 0.061) in univariate analysis and an approaching statistical trend (*P* = 0.054) in multivariate analysis.

**TABLE 4:** Univariate and multivariate logisitic regression analysis of factors associated with PC-AKI.

Variables	Univariate		Multivariate	
	OR (95% CI)	*P* Value	OR (95% CI)	*P* Value
Age	1.06 (0.96–1.16)	0.251	1.06 (0.95–1.18)	0.327
Male	0.33 (0.08–1.42)	0.136	0.61 (0.12–3.17)	0.553
BMI	0.98 (0.79–1.23)	0.874		
Smoker	0.75 (0.17–3.27)	0.704		
Alcoholism	1.83 (0.42–8.07)	0.422		
Diabetes	5.69 (1.10–29.3)	0.037	4.12 (0.74–23.06)	0.107
Hypertension	4.04 (0.48–33.79)	0.197	3.32 (0.35–31.38)	0.296
Potassium^a^ (mmol/L)	1.71 (0.28–10.52)	0.562		
Sodium^a^	0.92 (0.70–1.21)	0.540		
Chloride^a^	0.96 (0.78–1.19)	0.731		
Scr^a^	0.97 (0.92–1.02)	0.174		
Cys-C^a^	3.47 (0.25–48.43)	0.355		
NGAL^a^	1.02 (0.98–1.05)	0.371		
eGFR^a^	1.00 (0.94–1.07)	0.933		
Contrast dose	1.00 (0.99–1.01)	0.751	1.01 (0.99–1.02)	0.420
BRS	1.31 (0.02–1.10)	0.061	0.12 (0.02–1.03)	0.054

^a^ These variables indicating values before CAG/PCI.

Abbreviations: BMI, body mass index; BRS, Bicarbonate Ringer’s Solution; CAG, Coronary AngioGraphy; CI, confidence interval; Cys-C, Cystatin C; eGFR, estimated Glomerular Filtration Rate; NGAL, Neutrophil Gelatinase-associated ApoLipoprotein (NGAL); OR, odds ratio; PC-AKI, Post-Contrast Acute Kidney Injury; PCI, percutaneous coronary intervention; Scr, Serum Creatinine.

## 4 Discussion

Our study found that BRS might be more suitable than NS in preventing PC-AKI in patients with mild-to-moderate CKD who underwent CAG or PCI, without increasing the incidence of MACEs or RRT.

PC-AKI, also known as contrast-associated acute kidney injury (CA-AKI), is a specific type of kidney disease that occurs after exposure to contrast. The incidence of PC-AKI is about 2% in patients with normal renal function and 12%–27% in patients with CKD (McCullough et al., 1997; Azzalini et al., 2016). Participants in our study cohort were all with mild to moderate renal dysfunction, and the incidence of PC-AKI in the NS group was about 9.3%, which was consistent with previous studies.

To date, the pathogenesis of PC-AKI has not been clarified, and there are several possible mechanisms: (1) Direct damage effect of contrast agents on renal tubular epithelial cells induces cell apoptosis (McCullough et al., 2016; Heyman et al., 2008a). (2) The indirect effect of contrast is to reduce the perfusion of renal tissues, causing vasoconstriction, and result in decreased renal blood flow and hypoxic changes of renal tissues (Heyman et al., 2008b; McCullough et al., 2016). (3) Oxidative stress is another mechanism to cause PC-AKI, with the excessive reactive oxygen species to cause cell apoptosis (Mamoulakis et al., 2017).

Previous systematic reviews and meta-analyses have found a variety of factors associated with PC-AKI, such as female, CKD, diabetes, age >75 years, repeated exposure to contrast within a short period of time (48–72h), etc. (van der Molen et al., 2018b; Macdonald et al., 2022). Studies have confirmed that severe CKD is an independent risk factor for PC-AKI (Tsai et al., 2014; van der Molen et al., 2018b). Recent renal injury was related to the degree of renal function decline after angiography (Ramachandran and Jayakumar, 2020). Though other clinical factors have not been fully proved to be independent risk factors for PC-AKI, the incidence of PC-AKI was higher in patients with more concomitant risk factors than those with renal insufficiency only.

At present, there is no ideal treatment for patients with PC-AKI and hydration is the most important measure to prevent contrast-induced acute kidney injury. Although the cytological and molecular mechanism of hydration is still not clear, studies have found that hydration can increase urinary flow rate, reduce the concentration of contrast in renal tubules, and thus reduce the exposure time of contrast in renal tubular cells. Time-dependent toxic effects would be minimized in this way (Ellis and Cohan, 2009). Hydration could also reduce the release of vasoconstrictor hormones by inhibiting the activity of the renin-angiotensin system (Sterling et al., 2008). The renal tubular pressure is further reduced by decreased tubular-glomerular feedback, and the increased secretion of prostacyclin (Ellis and Cohan, 2009). Therefore, the benefits of hydration have been widely recognized, but the choice of hydration fluids is still inconclusive.

Several meta-analyses demonstrated that there were no evidence that oral hydration increased the risk of PC-AKI compared with intravenous route, however, these studies are limited by their heterogeneity and lack of robust clinical results (Hiremath et al., 2013; Cheungpasitporn et al., 2014; Agarwal et al., 2015). Intravenous hydration is still the recommended administrated route over oral pathways, because the latter is difficult to monitor and control bias. There are different intravenous hydration recommendations. The American College of Radiology guidelines recommend intravenous isotonic saline at 100 mL/h 6–12 h before until 4–12 h after angiography (Kodzwa, 2019). ESUR guidelines recommend 3 mL/kg/h of 1.4% bicarbonate 1 h before contrast exposure, followed by 1mL/kg/h over a period of 4–6 h, or 1mL/kg/h of 0.9% saline 3–4 h before and 4–6 h after exposure (van der Molen et al., 2018b). KDIGO guidelines recommend hydration at a rate of 1–1.5mL/kg/h for 3–12 h before contrast exposure, followed by 6–12 h of hydration with a target urine volume of >150 mL/h (Lameire and Kellum, 2013). Chinese consensus recommends intravenous hydration at a rate of 1.0 mL/kg/h 6–12 h before and 12–24 h after contrast exposure. However, long term hydration restricts patients’ mobility in perioperative rehabilitation training and is difficult to implement clinically, especially for emergency patients. And simplified rapid hydration has been proved to be non-inferior to standard hydration for CA-AKI prevention in patients with CKD (Liu et al., 2023), so we made the hydration regimen according to the ESUR guidelines.

Mueller’s study established the status of isotonic saline in hydration (Mueller et al., 2002). However, saline is a highly chlorinated fluid which may cause hyperchloremia, and a small randomized controlled test found that hyperchloremia was persistent in participants from the saline group, while not in those from plasma-Lyte 148 (a balanced crystalloid buffered with acetate) group (Chowdhury et al., 2012). Though some studies found that sodium bicarbonate solution was superior to NS (Merten et al., 2004; Masuda et al., 2007; Ozcan et al., 2007; Recio-Mayoral et al., 2007), the following studies could not get the same results (Adolph et al., 2008; Brar et al., 2008; Boucek et al., 2013). Hydration with bicarbonate did not significantly reduce the odds of CA-AKI in a meta-analysis of randomized controlled trials (Sharp et al., 2019). In addition, the sodium bicarbonate solution of high concentration may affect the acid-base balance, while the isotonic low concentration is complicated and time-consuming to prepare. Therefore, neither saline nor sodium bicarbonate was the ideal hydration fluid. BRS contains an appropriate amount of electrolytes and bicarbonate and is more closer to the normal physiological environment. It has been proved to prevent kidney injury in animal experiments (Pakfetrat et al., 2019), and postoperative renal injury in surgical patients (Wu et al., 2022; Yu et al., 2022). BRS also showed a potential renal function protective effect in critically ill patients, and a preventive effect on cisplatin-induced AKI in patients with esophageal cancer, compared with NS (Bian et al., 2022; Takemura et al., 2025). For the above reasons, we conducted this study and the results showed that BRS might be more suitable than NS in preventing PC-AKI.

Predictive models can be used for early screening of PC-AKI. The most widely used risk scoring model for patients underwent PCI was proposed by Mehran in 2004, which includes 8 variables (hypotension, intra-aortic balloon pump, congestive heart failure, chronic kidney disease, diabetes mellitus, age >75 years, anemia and contrast dose) (Mehran et al., 2004). This model was updated in 2021 (Mehran et al., 2021), but its application in the perioperative period is limited because it contains some operation-related factors (Mehran et al., 2019). Recently, a growing number of preoperative prediction models have also been proposed. For example, Liu et al. established a simple preoperative risk score, including age greater than 75 years, LVEF less than 40%, and Scr greater than 1.5 mg/dL, to predict the incidence of PC-AKI, and the results showed a similar prediction accuracy to that of Mehran score (Liu et al., 2015).

In addition to the rise in serum creatinine, alternative biomarkers have been proposed to predict PC-AKI, among which NGAL and Cys-C are the most concerned (Mahapatro et al., 2024). However, no statistical significance of NGAL and Cys-C was observed in this study. There might be several reasons. First, the optimal measuring timings of NGAL and Cys-C were at 4 h and 24 h after contrast use, respectively (Liu et al., 2012; Chen et al., 2020). These indicators were detected at 48 h after contrast exposure in our study, and they had decreased at that time due to the short half-life. Second, NGAL may not serve as a reliable biomarker in patients with CKD, since its measurement accuracy could be interfered with various factors including the presence of urinary protein, age, eGFR, etc (Wagener et al., 2008; Sahu et al., 2022).

Some preventive measures can be taken in the perioperative period, including: monitoring of Scr and urine volume within 72 h, choosing contrast with less impact on renal function (hypotonic or isotonic contrast), reducing the contrast doses without affecting the effect, and cease of diuretics and nephrotoxic drugs (such as metformin, aminoglycosides, non-steroids anti-inflammatory drugs) before CAG/PCI (van der Molen et al., 2018a; Zhang et al., 2020; Macdonald et al., 2022). The 2018 ESUR guidelines recommended maintaining the ratio of contrast dose (in grams of iodine) to absolute eGFR (in mL/min; corrected for body surface area) under 1.1 (Gurm et al., 2011; Kooiman et al., 2014), or the ratio of contrast volume (mL) to eGFR (ml/min/1.73 m^2^) under 3.0 when using an iodinated contrast at a concentration of 350 mg/mL (van der Molen et al., 2018a).

There were no MACEs or RRT required in our study cohort during the 30days follow-up. This could be explained by the following reasons. First, all patients received hydration in this study. Second, the enrolled patients had fewer risk factors and were classified as low and medium risk group of PC-AKI. Third, few patients with complex coronary artery lesions were enrolled in this study. Fourth, some slight symptoms might be ignored by the patients themselves, which were difficult to be observed and recorded since they were at home and not under the monitoring of researchers.

This study have several limitations. First, it was limited by its single-center design with a small sample size, making subgroup analysis difficult. Second, most patients were discharged within 2–3 days, only one creatinine result was available within the 48 h after CAG/PCI. Those whose creatinine peaked after 48 h were not included, which may have resulted in missed diagnoses of delayed-onset PC-AKI. Third, patients with severe cardiac and renal insufficiency were not included, and the efficacy and safety of hydration regimen in those patients were not evaluated. The results might not be applied to other populations. Fourth, the incidence of PC-AKI was low in the overall study cohort, which may affect the statistical significance. Thus, further evidence from well-designed multi-centre, large-sample studies are warranted to confirm these results.

## 5 Conclusion

This study demonstrates that BRS might be more suitable than NS in reducing the incidence of PC-AKI for patients with mild-to-moderate renal dysfunction who underwent CAG/PCI. However, further well-designed studies are warranted to confirm this.

## Data Availability

The original contributions presented in the study are included in the article/supplementary material, further inquiries can be directed to the corresponding authors.
